# Lipid rafts serve as signaling platforms for mGlu1 receptor-mediated calcium signaling in association with caveolin

**DOI:** 10.1186/1756-6606-7-9

**Published:** 2014-02-10

**Authors:** Seung-Eon Roh, Yun Hwa Hong, Dong Cheol Jang, Jun Kim, Sang Jeong Kim

**Affiliations:** 1 Department of Physiology, Seoul National University College of Medicine, 28, Yeongeon-dong, Jongno-gu, Seoul 110-799, Korea; 2 Department of Biomedical Science, Seoul National University College of Medicine, Seoul, Korea; 3 Neuroscience Research Institute, Seoul National University College of Medicine, Seoul, Korea; 4 Department of Brain and Cognitive Sciences, College of Natural Science, Seoul National University, Seoul, Korea; 5 Department of Biomedical Engineering, Huree University of Information and Communication Technology, Ulaanbaatar, Mongolia

**Keywords:** mGlu1α receptor, Lipid rafts, Caveolin, Calcium

## Abstract

**Background:**

Group I metabotropic glutamate receptors (mGlu1/5 receptors) have important roles in synaptic activity in the central nervous system. They modulate neuronal excitability by mobilizing intracellular Ca^2+^ following receptor activation. Also, accumulating evidence has indicated the association of Ca^2+^ signaling with lipid rafts. Caveolin, an adaptor protein found in a specialized subset of lipid rafts, has been reported to promote the localization of membrane proteins to lipid rafts.

**Results:**

In the present study, we investigated the role of lipid rafts on the mGlu1α receptor-mediated Ca^2+^ signaling in association with caveolin in hippocampal primary neurons and HEK293 cells. We show that the disruption of lipid rafts using methyl-β-cyclodextrin markedly decreased mGlu1α receptor-mediated Ca^2+^ transients and lipid rafts localization of the receptor. Furthermore, transfection of mGlu1α receptor with mutated caveolin-binding domain reduced localization of the receptor to lipid rafts. Also, application of a peptide blocker of mGlu1α receptor and caveolin binding reduced the Ca^2+^ signaling and the lipid rafts localization.

**Conclusions:**

Taken together, these results suggest that the binding of mGlu1α receptor to caveolin is crucial for its lipid rafts localization and mGlu1α receptor-mediated Ca^2+^ transients.

## Background

Metabotropic glutamate receptors (mGluRs) are members of the G protein-coupled receptor (GPCR) superfamily. They are activated by glutamate, which is a major excitatory neurotransmitter in the central nervous system (CNS) and regulate brain functions such as memory, motor control, and neuronal development [1]. mGluRs have been classified into three groups according to their sequence similarity, pharmacology and G protein coupling specificity [1,2]. Group 1 mGluRs, which encompass the mGlu1 and mGlu5 receptor, are expressed in several brain regions including the cortex, hippocampus and cerebellum [3]. They are selectively activated by the specific agonist, (*S*)-3,5-dihydroxyphenylglycine (DHPG) [4,5]. Exogenous activation by the agonist evokes an elevation of intracellular Ca^2+^ concentration, which contributes to the induction of long-term plasticity [6,7].

Regulation of neurotransmitter signaling has been found to be associated with lipid rafts, which are sphingolipid- and cholesterol-rich domains of the plasma membrane [8]. Several studies have shown that lipid rafts concentrate many of the regulators and ion channels involved in Ca^2+^ signaling, suggesting significant roles of lipid rafts in modulating Ca^2+^ signaling [9,10]. Lipid rafts exist abundantly in dendrites of neurons, in which they associate with glutamate receptors [11-15]. It has also been reported that the signaling of glutamate receptors is dependent on the integrity of lipid rafts. For instance, AMPA receptors localize to lipid rafts, and their residency in rafts is regulated by the NO-mediated signaling pathway [13]. Also, the NMDA receptor is associated with lipid rafts, and their interaction is related to the signaling of NMDA-induced neuronal death [11].

Also, it was found that mGluRs co-localize in lipid rafts together with caveolin [16,17]. We have previously reported that mGlu1α receptor interacts with caveolin, a scaffolding protein found in a specialized subset of lipid rafts, which mediates the agonist-induced internalization of receptor [16]. Furthermore, it has been reported that caveolin knockout mice exhibit impaired mGluR-dependent long-term depression (LTD) at CA3-CA1 synapses of the hippocampus [18]. Although lipid rafts have been extensively reported to regulate glutamate receptors, little is known about their contribution to the regulation of glutamate receptor Ca^2+^ signaling.

In the present study, we investigated whether the integrity of lipid rafts is involved in mGlu1 receptor-mediated Ca^2+^ signaling and also whether it affects localization of the receptor to lipid rafts. To this end, we performed Ca^2+^ imaging with the mGlu1 receptor agonist and examined the co-localization of mGlu1 receptor with lipid rafts using a cholesterol extraction drug. We also examined whether the interaction between mGlu1 receptor and caveolin affects mGlu1 receptor-mediated Ca^2+^ signaling and the lipid rafts localization by disrupting binding sites using mutant transfection or peptide blockade.

## Results

### Disruption of lipid rafts impairs mGlu1α receptor-induced Ca^2+^ signaling and lipid rafts localization of mGlu1 receptor in hippocampal neurons

**Figure 1:**
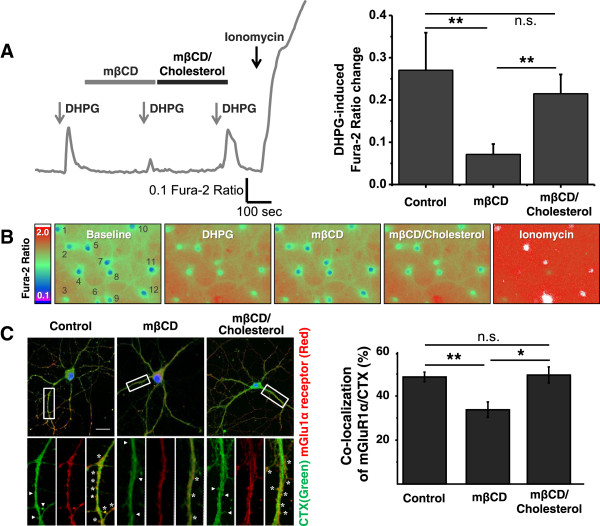
**Disruption of lipid rafts decreases mGlu1 receptor-induced Ca**^**2+ **^**transient and receptor localization to lipid rafts in hippocampal neurons. (A)** A representative trace of Fura-2 Ca^2+^ imaging is shown. Cells loaded with Fura-2/AM in NT solution for 30 min were stimulated with 50 μM DHPG during 60s (arrows) in the presence of MPEP (10 μM) to block mGlu5 receptor activation. DHPG was also given after treatment of mβCD (2 mg/ml for 300 sec) for cholesterol depletion and mβCD/cholesterol complex for cholesterol replenishment (10 μM for 300 sec) as well. Gray and black bars represent treatment of mβCD and mβCD/cholesterol complex. Population data are shown in right panel. n=33. **(B)** Representative field images from Ca^2+^ imaging of A. Images are presented, left to right: Baseline, imaging field of baseline. DHPG, at the peak ratio by applying DHPG. mβCD and mβCD/Cholesterol, the peak in the presence of mβCD and mβCD/Cholesterol. Cells analyzed are numbered in the baseline image. Scale bar for Fura-2 ratio is shown left. **(C)** Confocal images visualizing co-localization of mGlu1α receptor (red) and lipid rafts (green) following mβCD and mβCD/cholesterol complex. White boxes in the upper figures are enlarged in lower figures. Overlaying areas (yellow) are designated with asterisks, implying co-localization of mGlu1α receptor to CTX-Alexa 488. Spines are indicated with white arrow heads. Co-localization scores are presented in right panel. n = 11 (Control), 12 (mβCD), and 15 (mβCD/cholesterol complex). Scale bar = 10 μm. The data is shown as mean ± SEM. ANOVA followed by post hoc test, n.s., non-significant, *, P < 0.05, **, P < 0.001.

To investigate the role of lipid rafts in mGlu1 receptor functionality, we first asked whether the disruption of lipid rafts affects mGlu1 receptor-induced Ca^2+^ transients. For this purpose, we performed Ca^2+^ imaging with hippocampal primary neurons using a ratiometric Ca^2+^ dye, Fura-2/AM in normal Tyrode’s solution (NT solution) using a lipid rafts disturbing drug. Since hippocampal cultured neurons express both mGlu1 and 5 receptors [19,20], we always measured cytosolic Ca^2+^ level ([Ca^2+^]_c_) in the presence of 2-Methyl-6-(phenylethynyl)-pyridine (MPEP, 10 μM), the selective antagonist of mGlu5, to specifically observe mGlu1 receptor-mediated responses. As shown in Figure 1A, application of DHPG (50 μM) for 60s robustly induced [Ca^2+^]_c_ increase. To disrupt lipid rafts, cells were perfused with methyl-β-cyclodextrin (mβCD), a drug able to solubilize and actively sequester cholesterol from membranes as previously described [21-23]. Bath-application of 2 mg/ml mβCD for 300 sec was sufficient to dramatically impair DHPG-induced Ca^2+^ influx (control: 0.27 ± 0.088, mβCD: 0.071 ± 0.025, P < 0.001 compared to control). Meanwhile, the amplitude of the first and second DHPG-induced Ca^2+^ increase in the absence of mβCD were not significantly different, excluding a possible tachyphylaxis to DHPG in our system (Data not shown). Next, to reverse the effect of mβCD, we used mβCD (2 mg/ml)/cholesterol (10 μg/ml) complex which was reported to attenuate mβCD-mediated lipid rafts damage by restoring cholesterol content on plasma membrane [24,25]. As shown in Figure 1A, after 300 sec perfusion of mβCD/cholesterol complex, DHPG-induced Ca^2+^ response was significantly recovered (0.215 ± 0.046, P < 0.001 compared to mβCD). Also, it was not found to be significantly different compared to control (P = 0.204), clearly validating the efficacy of the drug. Taken together, the data clearly suggest that the integrity of lipid rafts is crucial for mGlu1 receptor-mediated Ca^2+^ signaling, as additionally illustrated by representative images of Fura-2 Ca^2+^ imaging field (Figure 1B).

To further investigate how lipid rafts are involved in the mGlu1 receptor activity, we examined the lipid rafts localization of the receptor in the presence of mβCD or mβCD/cholesterol by confocal microscopy. We used Alexa 488-conjugated cholera toxin B subunit (CTX-Alexa 488, 2 μg/ml) to label ganglioside GM1, a well-known marker of lipid rafts [26]. To exclude the possible signal contamination of endocytosed CTX and mGlu1 receptor, cells were stained after fixation. Given the validation of antibody specificity of anti-mGlu1α receptor (Additional file 1: Figure S1), we tested the efficacy of GM1 labeling by CTX-Alexa 488 by simultaneously staining hippocampal neurons with CTX and anti-GM1 antibody, and observed almost identical expressions of both throughout soma and dendrites. This result contrasts with transferrin receptor, a non-lipid rafts marker, which was not co-localized with CTX-labeled regions (Additional file 1: Figure S1B). Double-labeling of CTX and mGlu1α receptor revealed that mGlu1α receptor co-localizes with lipid rafts throughout the cells including spines (Figure 1C, control: 48.7 ± 2.2%). Upon incubation with 2 mg/ml mβCD treatment, the co-localization was significantly decreased (Figure 1C, mβCD: 33.7 ± 9.3%, P < 0.01 compared to control). However, mβCD/cholesterol complex treatment did not affect the co-localization of mGlu1 receptor with lipid rafts (mβCD/cholesterol: 49.5 ± 9.7%, P = 0.208 compared with control and P < 0.05 compared with treated mβCD). Present results indicate that the integrity of lipid rafts affects the co-localization with mGlu1 receptor. Collectively, as we observed disturbance of both lipid rafts localization and Ca^2+^ signaling by mβCD, these data imply the association of lipid rafts localization of mGlu1 receptor with agonist-induced Ca^2+^ transients of the receptor.

### Binding of mGlu1α receptor and caveolin affects lipid rafts-targeting of the receptor

**Figure 2:**
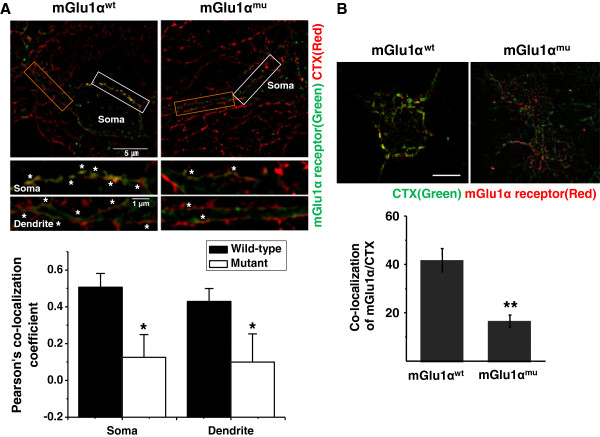
**Mutation of caveolin binding site of mGlu1 receptor induces its lipid rafts localization.** Hippocampal primary neurons were transfected with SEP-mGlu1α receptor^wt^ (wild-type) and SEP-mGlu1α receptor^mu^ (mutant) constructs. After staining with CTX-Alexa 488, images were acquired with super-resolution microscopy, N-SIM. **(A)** White and orange boxes in upper images for soma and primary dendrites, respectively, are enlarged at the bottom. Overlapping areas (yellow) were indicated with asterisks, implying co-localization of mGlu1α receptor to CTX-Alexa 488. Upper and lower scale bars represent 5 μm and 1 μm. Co-localization values are obtained by Pearson’s correlation method. Values represent Pearson’s co-localization coefficient ± SEM. n = 11 (mGlu1α^wt^) and 6 (mGlu1α^mu^). Student’s t test, * P <0.05. **(B)** TIRF images visualizing co-localization of RFP-mGlu1α receptor wild-type (mGlu1α^wt^) or RFP-mGlu1α receptor mutant (mGlu1α^mu^) and lipid rafts (green). HEK293 cells were transfected with RFP-mGlu1α^wt^ or - mGlu1α^mu^ and labeled with CTX-Alexa 488 (green). Overlaying areas (yellow) indicate co-localization of mGlu1 receptorα with CTX-Alexa 488. n = 18 (mGlu1α^wt^) and 18 (mGlu1α^mu^). Scale bars = 10 μm. Co-localization scores are presented as average values ± SEM. Student’s t test, **, P < 0.01.

We have previously described that agonist-induced internalization of mGlu1α receptor is mediated by the association with caveolin, an adaptor protein found in a subset of specialized lipid rafts. Also, the binding was abolished by mβCD, which, in turn, also blocked the agonist-induced internalization of mGlu1α receptor [16]. Hence, we asked whether interference of the interaction affects lipid localization of mGlu1α receptor. To this end, we used mGlu1α receptor constructs of wild-type (mGlu1α^wt^) and mutant with disrupted caveolin binding sites (mGlu1α^F609,614A^: mGlu1α^mu^), which is reported to impair agonist-induced Ca^2+^ transients [16]. These constructs were fused with super-ecliptic pHluorins (SEP), the pH-sensitive variant of GFP, to exclusively visualize mGlu1α receptors on the cell surface. For ultra-fine illustration of the localization, we utilized super-resolution Structured Illumination Microscopy (SIM, Nikon). Hippocampal primary neurons were transfected with SEP-mGlu1α^wt^ or SEP-mGlu1α^mu^ and simultaneously stained with CTX-Alexa 594. SIM imaging and Peason’s correlation (PC) analysis clearly revealed the significant decrease of co-localization of SEP-mGlu1α^mu^ with lipid rafts compared to SEP-mGlu1α^wt^ at the soma (mGlu1α^wt^: 0.557 ± 0.079 Pearson’s co-localization coefficient, mGlu1α^mu^: 0.125 0 0.123 Pearson’s r, P < 0.05) and at dendrites (mGlu1α^wt^: 0.415 ± 0.097 Pearson’s co-localization coefficient, mGlu1α^mu^: 0.099 ± 0.153 Pearson’s co-localization coefficient, P < 0.05) (Figure 2A). The data clearly indicate that the caveolin binding site mutation abolishes lipid rafts targeting of mGlu1α receptor.

To complementarily corroborate the present results, we executed co-localization analysis with Total Internal Reflection Fluorescence (TIRF) imaging to specifically visualize the cell surface in HEK293 cells. HEK293 cells were imaged after transfection with RFP-mGlu1α^wt^ or -mGlu1α^mu^ constructs and lipid rafts labeling by CTX-Alexa 488. The result indicated a significant reduction of co-localization between mGlu1α^mu^ and lipid rafts compared to mGlu1α^wt^ (Figure 2B, mGlu1α^wt^: 42.2 ± 4.8% and mGlu1α^mu^: 17.1 ± 2.6%, P < 0.01). In conclusion, these results suggest that the interaction of mGlu1α receptor with caveolin is crucial for the lipid rafts localization of the receptor.

### Blocking the interaction of mGlu1α receptor and caveolin decreases mGlu1α receptor -induced Ca^2+^ signaling and receptor localization to lipid rafts in hippocampal neurons

**Figure 3:**
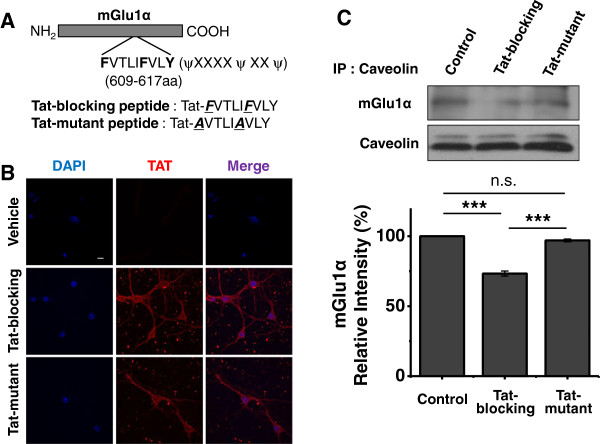
**Design of Tat-peptides for blocking mGlu1 receptor-caveolin interaction and its efficacy. (A)** Fusion synthetic peptides encoding Tat-blocking peptide and Tat-mutant peptide are shown. Tat-mutant peptide was generated by a dual amino acid mutation (underlined) in the caveolin binding motif (609-617aa). aa, amino acid. X and ψ indicate any amino acid and aromatic amino acid, respectively. **(B)** Validation of incorporation of Tat-blocking peptide/mutant peptides into hippocampal primary neurons. Cells incubated with both peptides for 45 mins were stained with anti-Tat antibody and imaged with confocal microscopy. The small dots stained with anti-Tat antibody are cell debris that may not affect our analytic measurements with co-localization study and Ca^2+^ imaging. The scale bar indicates 5 μm. **(C)** Co-immunoprecipitation assay of caveolin and mGlu1 receptor in hippocampal primary neurons after incubation with both peptides for 45 mins. Cell lysates were immunoprecipitated with anti-caveolin antibody and immunoblotted with anti-mGlu1 receptor antibody. Quantification was shown below. The values represent relative average intensity normalized by control value ± SEM. ANOVA with posthoc test, n.s., non-significant, ***, P < 0.001 (n=3).

Now that we have confirmed the importance of lipid rafts integrity and of the mGlu1α receptor-caveolin interaction in the functionality of mGlu1α receptors, we next investigated whether mGlu1 receptor-induced Ca^2+^ signaling requires the interaction with caveolin in cultured hippocampal neurons. Since hippocampal neurons endogenously express mGlu1α receptors and caveolin, we treated cells with synthetic peptides consisting of the caveolin binding motif of the mGlu1α receptor to abolish the interaction. The peptide sequence was used from our previous study which describes the amino acid sequence of the caveolin binding domain of the mGlu1α receptor [16]. The peptide was made cell-permeable by attaching a cell-penetrating peptide (CPP), human immunodeficiency virus-type 1 Tat sequence (YGRKKRRQRRR). We generated Tat-blocking peptide (Tat-FVTLIFVLA) for interfering with the interaction and Tat-mutant peptide with dual point mutations (Tat-*A*VTLI*A*VLA) as a negative control (Figure 3A). First, we demonstrated successful incorporation of both peptides into the hippocampal primary neurons by staining with anti-Tat antibody, as Tat peptides were found to exist throughout the cells including plasma membranes (Figure 3B). Next, as revealed by co-immunoprecipitation (Co-IP) assay in Figure 3C, preincubation of Tat-blocking peptide was shown to significantly reduce the interaction between mGlu1α receptor and caveolin when immonoprecipitated with anti-caveolin antibody in hippocampal primary neurons (Tat-blocking peptide: 73.3 ± 1.8% of control, P < 0.001 compared to control), while Tat-mutant peptide incubation did not (Tat-mutant peptide: 97.0 ± 1.0% of control, P = 0.369 compared to control, P <0.001 compared to Tat-blocking peptide). The efficacy of Tat-blocking peptide was also demonstrated by immunoprecipitating with anti-mGlu1α receptor antibody in HEK293 cells after overexpression of mGlu1α receptor (Additional file 1: Figure S2A).

**Figure 4:**
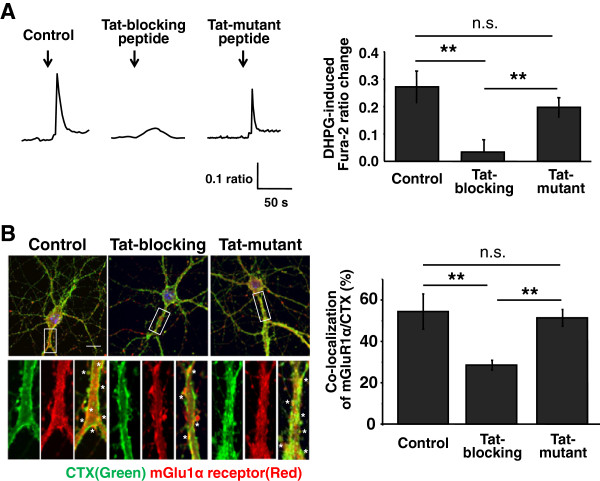
**Tat-blocking peptide causes reduction in mGlu1 receptor-mediated Ca**^**2+ **^**transient and its lipid rafts localization in hippocampal neurons. (A)** Representative traces of the Fura-2 Ca^2+^ imaging with Tat-peptides. Cells were incubated with each peptide (10 μM for 45 min) before bath application of DHPG (50 μM, for 60s) as indicated by arrows, in the presence of 10 μM MPEP. Population data are shown in right panel. n = 12 (Control), 15 (Tat-blocking peptide), 13 (Tat-mutant peptide). **(B)** Confocal images visualizing co-localization of mGlu1α receptor and lipid rafts treated with Tat-peptides. Cells were labeled with CTX-Alexa 488 (green) and anti-mGlu1α receptor antibody (red). White boxes in the upper images are enlarged in lower panels. Overlapping areas (yellow) indicate co-localization of mGlu1α receptor to CTX-Alexa 488. n = 21 (Control), 20 (Tat-blocking peptide), 21 (Tat-mutant peptide). Scale bars = 10 μm. The data is shown as mean ± SEM. An ANOVA with posthoc analysis was performed. n.s., non-significant, **, P < 0.01.

Since the efficacy of the Tat-blocking peptide was validated, we examined whether the peptide impairs mGlu1 receptor-induced Ca^2+^ transients in hippocampal neurons. Here, we also performed Ca^2+^ imaging in the presence of the selective antagonist of mGlu5 receptor, MPEP (10 μM) to specifically observe mGlu1 receptor-induced responses. As shown in representative [Ca^2+^]_c_ traces and ratio change quantification of Figure 4A, application of Tat-blocking peptide (10 μM) for 45 min was sufficient to markedly reduce mGlu1 receptor-induced Ca^2+^ transients (control: 0.28 ± 0.08 ratio, Tat-blocking peptide: 0.02 ± 0.01 ratio, P < 0.01). However, the amplitude was not significantly decreased when incubated with Tat-mutant peptide (Tat-mutant peptide: 0.20 ± 0.05 ratio, P < 0.01 compared to control, P = 0.379 compared to control). A similar result was obtained in HEK cells transfected with mGlu1α receptor (Additional file 1: Figure S2B). Taken together, these results indicate that blockage of interaction of mGlu1α receptor and caveolin impairs agonist-induced Ca^2+^ signaling of mGlu1 receptor.

To examine whether the Tat-blocking peptide is also responsible for disrupting lipid rafts localization of the receptor in hippocampal neurons, we performed double-labeling immunocytochemistry of endogenous mGlu1α receptor and lipid rafts. Following 45 min treatment of Tat-blocking or -mutant peptide (10 μM), cells were stained with CTX-Alexa 594 and anti-mGlu1α receptor antibody. As shown in Figure 4B, Tat-blocking peptide significantly reduced the localization of the mGlu1α receptor to lipid rafts (control: 54.4 ± 8.5%, Tat-blocking peptide: 28.5 ± 4.7%, P < 0.01), indicating the importance of the interaction of both proteins in lipid rafts localization of mGlu1α receptor. Tat-mutant peptide had no noticeable effect on the localization of the receptor to lipid rafts (Tat-mutant peptide: 51.3 ± 7.0%, P = 0.38 compared to control, P < 0.01 compared to Tat-blocking peptide). In conclusion, these data strongly suggest that the interaction of mGlu1α receptor and caveolin is critical for lipid rafts localization and agonist-induced Ca^2+^ signaling of the endogenous mGlu1α receptor.

## Discussion

In the present study, we provide a series of evidences for the importance of lipid rafts as a signaling platform for mGlu1α receptor. Using multiple measures, we demonstrated that the integrity of lipid rafts, localization of mGlu1 receptor to lipid rafts and the interaction with caveolin are crucial for agonist-induced mGlu1 receptor Ca^2+^ transients. First, we observed that the mβCD compromised Ca^2+^ signaling of mGlu1 receptors and significantly reduced its co-localization with lipid rafts in hippocampal primary neurons. We also showed the interaction with caveolin is necessary for lipid rafts localization using mGlu1 receptor constructs with mutated caveolin binding sites in hippocampal primary neurons and HEK293. The application of a blocking peptide for binding between caveolin and mGlu1 receptor further supports that such interaction plays an important role in locating mGlu1 receptors to lipid rafts and in its Ca^2+^ signaling in hippocampal primary neurons. Therefore, we suggest that lipid rafts, in association with caveolin, serves as a signaling microdomain and is a critical requirement for mGlu1 receptor-mediated signal transduction.

Intracellular Ca^2+^ is a second messenger that controls many cellular processes, including neuronal excitability, synaptic plasticity and neuronal death [27-29]. Intracellular Ca^2+^ homeostasis is tightly regulated and disturbances in Ca^2+^ homeostasis have been implicated in several neurodegenerative diseases [30,31]. It is not at all surprising that disturbances in Ca^2+^ signaling pathways underlie neuronal loss, since many factors involved in neuronal function are dependent on Ca^2+^ signaling [32]. Although lipid rafts regulate Ca^2+^ signaling in cardiac myocytes and other tissues, this is yet to be well established in the nervous system. A recent study in astrocytes shows several proteins that form the inositol 1,4,5-triphosphate (IP_3_) dependent Ca^2+^ cascade – metabotropic receptor P2Y1, Gq, IP_3_ receptor (IP3R), phospholipase C β (PLCβ) and protein kinase Cα (PKCα) – are all enriched in lipid rafts. Stimulation of cells with a purinergic agonist recruited PLCβ and PKCα to rafts fractions, whereas lipid raft disruption showed inhibition of agonist-evoked Ca^2+^ waves [33]. It has also been reported that various membrane receptors localize in lipid rafts and their signaling is dependent on lipid raft integrity in nervous systems [34-38]. Although lipid rafts regulate glutamate receptor signaling, little is known about their contribution to the Ca^2+^ signaling of glutamate receptors. This study shows that lipid rafts regulate Ca^2+^ signaling of glutamate receptor in nervous system.

One hypothesis to explain the decrease in mGlu1α receptor-induced Ca^2+^ transients depending on lipid raft availability is the reduced interaction of Ca^2+^ signaling proteins [34-36,38]. Well-established pathways of group I mGluR Ca^2+^ signaling are the G-protein-dependent (Gαq) intracellular Ca^2+^ release via PLC and IP_3_ activation pathway and the Ca^2+^ influx via transient receptor potential canonical (TRPC) channel pathway. Interestingly, not only group I mGluRs but also Gq/11, PLC, IP3R, and TRPC, are now known to be present in lipid raft domains [34-36,38]. Thus, it is conceivable that the co-localization of mGlu1α receptors and their Ca^2+^ signaling partners, facilitated by their residence in lipid rafts, might regulate coupling of the receptor signaling. It would be interesting further study to examine the function of these molecules after impairing the lipid rafts integrity. A different hypothesis is that mGluRs exist in different affinity states for glutamate, depending on the membrane composition. The receptor is in a high-affinity state when associated with sterol-rich lipid rafts, and in a low-affinity state outside of rafts [39]. Enrichment of the membranes with cholesterol shifts the receptor into the high-affinity state, and induces its association with rafts. It is also possible that differences of agonist binding affinity according to differential lipid raft localization might regulate the coupling of the receptor signaling. Another recent study suggests that mGlu1α is recruited by agonist to lipid rafts and this is supported in part by intact cholesterol recognition/interaction amino acid consensus (CRAC) motif [40]. The data are in line with our result which showed impairment of Ca^2+^ signaling by cholesterol extracting drug, mβCD, implying the importance of cholesterol in the receptor function. Further, several studies have suggested MAPK (ERK1/2) and PI3K-Akt-mTOR pathways as down-stream effectors of mGlu1 receptor activation which are involved in long term depression (LTD) [17,41]. It might be interesting to investigate whether mβCD or caveolin binding blockade could affect the activation of these pathways, ultimately influencing synaptic plasticity.

But the function of mGlu1 receptor outside the lipid rafts is still unknown. At least, however, we propose that mGlu1 receptors within lipid rafts display higher activity than those outside lipid rafts as revealed by our data (Figure 1). It is also supported by the previous report which shows transition from resting state to active state of ligand binding domain when the receptor moves to lipid rafts [39]. Also, it may be explained that the mGlu1 receptors may have redundancy in order that only a small fraction of receptors remain active, leaving resting molecules ready to instantly participate in responses to external stimuli. Hence, the activity control by lipid rafts localization might be thought as an important mechanism for mGlu1 receptor function.

Previously, mechanisms of caveolin recruiting receptors to lipid rafts have been proposed [42,43]. As shown through *in vitro* studies, caveolin is sufficient to recruit soluble Ras, the class of signal transducing GTPase, onto lipid membranes [43]. In our previous study, we showed that mGlu1α receptor contains the putative caveolin binding motifs spanning from the first transmembrane domain to the first inner loop, and from the seventh transmembrane domain to the C-terminal domain of the receptor In HEK293 cells, mGlu1α receptor mutant which has mutations in only the first transmembrane domain sufficiently reduced lipid rafts localization of receptor. In hippocampal neurons, we also showed that a blocking peptide impairs the interaction of mGlu1α receptor with caveolin, resulting in a disruption to the lipid raft localization of the receptor. Incubation time (45 min) of the blocking peptide was sufficient to interrupt the interaction between receptor and caveolin, considering the rapid constitutive recycling of mGlu1α receptor [17]. A key finding in this study is that interactions between mGlu1α receptor and caveolin are necessary for lipid rafts localization of the receptor and these ultimately form the Ca^2+^ signaling pathway. As for the seemingly discrepancy of the effect of Tat-blocking peptide on the Ca^2+^ transient and immunoprecipitation of mGlu1 receptor, it is considered that the Tat-blocking peptide significantly perturbs the receptor function while retaining some part of the interaction between mGlu1 receptor and caveolin. At any rate, our data are consistent with a study which showed that ATP-induced Ca^2+^ increases originated in specific areas of the caveolin-enriched plasma membrane in endothelial cells, suggesting that caveolin may be involved in the initiation of agonist-stimulated Ca^2+^ signaling [44]. However, another report suggests that the interaction of mGlu1 receptor and caveolin-1 is not required for its localization to lipid rafts [40]. It showed that mGlu1 receptor mutated with two caveolin-1 binding sites displayed comparable agonist binding affinity and agonist-induced localization to lipid rafts with wild-type. However, since the mutant itself has significantly reduced surface expression as they discussed, it is difficult to directly compare these measures between wild-type and mutant, while we used a mutant for one caveolin binding site which has normal surface expression level. Also, the experimental conditions such as cell type and agonist are different with ours. Another *in vivo* result from caveolin^-/-^ brain cortex containing glial tissues does not directly implicate it as neuronal tissues. Still, caveolin should be functionally involved with mGlu1 receptor since caveolin knockout mice show impaired mGlu receptor LTD [18]. Overall, since we observed decreased calcium transients when using mutant mGlu1 receptor [16] and when treated with caveolin binding blocking peptides in our present study, we suggest that caveolin affects, rather than agonist binding affinity of mGlu1 receptor, lipid rafts localization and coupling with downstream effector.

mGlu1 receptor has been implicated in several neurological disorders. For example, disturbances in Ca^2+^ homeostasis in hippocampal cells have been implicated in neurodegenerative diseases such as Alzheimer’s disease (AD) [45]. Specifically, the impairment of declarative memory coincides with the extracellular accumulation of amyloid-β protein (Aβ) [46] and Aβ-enhanced LTD is mediated by mGluR activity and requires an influx of extracellular Ca^2+^[47]. It has been shown that enhanced mGluR signaling and Ca^2+^ release regulated by IP3R were identified as underlying causes of the age-dependent cognitive phenotypes observed [48]. Also, chronic pain is a disease caused by plasticity changes in synapses of nociceptive center and such process is mediated by mGluRs including mGlu1 receptor [49]. Indeed, antagonism of the receptor has emerged as a potential treatment target of pain [50]. Further, there are several evidence which implicate mGlu1 receptor in ataxia [51,52], and anxiety [53,54]. As such, our findings hold significance in that manipulation of lipid rafts and caveolin binding sites which substantially affect the mGlu1 receptor function could modulate the states of diseases described above.

## Conclusions

In conclusion, the findings described here suggest that lipid rafts regulate Ca^2+^ signaling of mGlu1 receptors, and caveolin is required for receptor residency in lipid rafts, suggesting lipid rafts and caveolin as modulation targets for related diseases.

## Methods

### Cell culture, transfection and DNA constructs

Primary hippocampal neurons were prepared from postnatal day 1 C57BL/6 mouse. In brief, hippocampi were isolated, stripped of meninges, and enzymatically dissociated with trypsin (Invitrogen, USA). After washing, cells were plated on 12 mm coverslips (0.5 × 10^4^ cells) for intracellular Ca^2+^ measurement or immunocytochemistry. Cultures were incubated at 5% CO_2_ and 37°C with Neurobasal media (Invitrogen, CA, USA) supplemented with B27 (Invitrogen) and 0.5 mM glutamine (Invitrogen). At DIV3, cells were treated with 1-β-D-Arabinofusylcytosine (5 μM, Calbiochem, USA) and fed twice a week with new media until DIV10-14 for experiments including Ca^2+^ imaging, microscopy and western blotting. The use and care of animals employed in this study followed the guidelines of the National Institutes of Health Animal Research Advisory Committee. We have followed ARRIVE (Animal Research: Reporting *In Vivo* Experiments) guidelines.

Human embryonic kidney 293 (HEK293) cells were grown in Dulbecco’s Modified Eagle Medium (Invitrogen) supplemented with 10% (v/v) fetal bovine serum and 1% antibiotics (Invitrogen). For the transient expression of mGlu1α receptor, cells growing on 12 mm cover slips or in 35 mm dishes were transfected with 0.5 or 3 μg of DNA, respectively, using FuGENE 6 transfection reagent (Roche Molecular Biochemicals, IN, USA) according to manufacturer’s instructions. Wild-type receptor (mGlu1α receptor^wt^) DNA constructs containing red fluorescent protein (RFP-mGlu1α receptor in pRK5 vector) in the extracellular N-terminus were used as previously described [16]. Mutants of mGlu1α receptor (mGlu1α receptor^mu^; mGlu1α receptor^F609,614A^) were generated using the QuikChanges site-directed mutagenesis kit (Stratagene, La Jolla, CA, USA) following the manufacturer’s instructions, using forward primer (22 mer): 5′-ctcgtgacgc tggccgtcac cctcatcgcc gttctgtacc gg-3′ and reverse primer (22 mer): 5′ccggtacaga acggcgatga gggtgacggc cagcgtcacg ag-3′. These constructs were further fused with super ecliptic pHluorin (SEP), the pH-sensitive variant of GFP, for SIM imaging.

### Antibodies

Rabbit anti-mGlu1α receptor was obtained from Dr. C. H. Kim (Department of Pharmacology, Yonsei University College of Medicine, Korea). Rabbit anti-caveolin, rabbit anti-transferrin receptor and mouse anti-mGlu1α receptor were purchased from BD Bioscience (Lexington, KY, USA). Mouse monoclonal anti-Tat was purchased from Immuno Diagnostics (Woburn, MA, USA). Alexa 488 conjugated cholera toxin B subunit, Alexa 594 conjugated secondary antibodies, and rabbit anti-monosialotetrahexosylganglioside (GM1) were purchased from Molecular Probes (Carlsbad, CA, USA). An appropriate horseradish-peroxidase-conjugated goat IgG as secondary antibody (Stressgen, Collegeville, PA, USA) was immunoblotted.

### Intracellular Ca^2+^ measurements

Cells on coverslips were loaded for 30 min at 37°e with acetoxy-methyl-ester Fura-2 (Fura-2/AM; Molecular Probes, CA, USA) in normal Tyrode’s solution (NT; 140 mM NaCl, 5 mM KCl, 2 mM CaCl_2_, 1 mM MgCl_2_, 10 mM glucose, and 10 mM HEPES, pH 7.35) supplemented with 0.01% pluronic acid (Sigma, USA). Ca^2+^ imaging experiments were performed using a microscope (Olympus BX50) with 40 × UV objective (Olympus, Tokyo, Japan). For Fura-2/AM excitation, a monochromator polychrome-II (TILL-Photonics, Munich BioRegio, Germany) was controlled by Axon Imaging Workbench software 6.0 (AIW; Axon Instruments, CA, USA) to provide sequential illumination at two alternating wavelengths, 340 and 380 nm. Fluorescence of Fura-2/AM was detected at an emission wavelength of 510 nm. Video images were acquired using an intensified CCD camera (LUCA; Andor Technology, Belfast, UK). Fluorescence emission ratios following excitation at 340 and 380 nm were calculated. The values were exported from AIW to Origin Pro 8.0 software (OriginLab, MA, USA) for additional analysis and plotting. After randomly selecting an imaging field, all the individual cells in the field were selected as ROI, 10-15 cells each. All experiments were independently performed at least three times.

### Immunocytochemistry and cholera toxin (CTX) cell-surface labeling

Cholera toxin B subunit which binds specifically to ganglioside GM1 was used as marker of surface lipid rafts. Following a rinse with PBS (137 mM NaCl, 2.7 mM KCl, 10 mM Na_2_HPO_4_ • 2 H_2_O, and 2.0 mM KH_2_PO_4_, pH 7.4), cells were fixed with 4% paraformaldehyde (PFA) and incubated with 2 μg/ml Alexa 488 or 594 conjugated cholera toxin B subunit (CTX-Alexa 488 or 594) in PBS at room temperature for 15 min. Cells were then washed with PBS and further fixed with 4% paraformaldehyde (PFA) for 30 min at room temperature. For double labeling of the lipid rafts makers and mGlu1α receptor, neurons were permeabilized with 0.2% saponin for 10 min at RT and blocked with 5% normal goat serum for 1 h at RT. Rabbit anti-mGlu1α receptor was exposed to neurons overnight at 4°C. Neurons were then treated with anti-rabbit Alexa 594 conjugated secondary antibody for 45 min at RT and mounted on slides using ProLong Gold Anti-fade reagent (Invitrogen). Images for mGlu1α receptor co-localization with lipid rafts markers were acquired by Olympus FV-1000 confocal microscope (Olympus, Japan) equipped with 100× oil-immersion lens (1.35 NA). Cells were excited with 488 nm (from an argon laser) and 559 nm light (from a diode laser).

### Co-localization analysis

Co-localization of mGlu1α receptor to CTX-Alexa 488 was analyzed in the soma and dendrites up to 50 μm away from the soma of the neurons. Images were acquired from 3-4 dendrite branches per neuron from several neurons in each condition, and the co-localization scores were obtained as the percentage of overlapping pixels of CTX with mGlu1 receptor or other proteins including flotillin, caveolin, and transferrin receptor after exclusion of background fluorescence. We manually selected soma or dendritic regions of interest for analysis and subtracted the mean intensity of background, outside of soma or dendrites. Co-localization was generally quantified using the MetaMorph 6.0 software (Molecular Devices, Downingtown, PA, USA). For more detailed analysis of co-localization accordingly with SIM imaging in Figure 2, the analysis was performed using Pearson’s correlation test by Nikon NIS-Element-AR software (Nikon, Japan) and data was presented as Pearson’s correlation coefficient, where the coefficient between 0 and -1 implies no co-localization, whereas a value 1 corresponds a perfect co-localization. The specificity of lipid rafts and mGlu1 receptor staining was tested by negative control staining, transferrin and IgG, respectively (Additional file 1: Figure S1).

### Structured illumination microscopy

To analyze the co-localization of mGlu1 receptor and lipid rafts in hippocampal primary neurons, we utilized the super-resolution structured illumination microscopy (SIM; Nikon N-SIM). Images were obtained by Eclipse Ti-E inverted microscope equipped with Nikon’s legendary CFI Apo TIRF 100× oil objective lens (NA 1.49) and iXon DU-897 EMCCD camera (Andor Technology). Specimens were excited with a diode laser (488 nm and 561 nm) and acquired images were processed with deconvolution using NIS-Element-AR software (Nikon).

### Total internal reflection fluorescence (TIRF) imaging

TIRF imaging was performed to precisely observe cell surface proteins according to our previous procedures [16]. In brief, RFP-mGlu1α receptor^wt^ or -mGlu1α receptor^mu^ expressing HEK cells were washed with PBS and stained with CTX-Alexa 488. Cells were then washed with PBS, fixed with 4% PFA for 30 min at room temperature and mounted on slides using ProLong Gold Anti-fade reagent (Invitrogen). Imaging was performed using Olympus IX-71 inverted microscope fitted with a X60, 1.45 N.A. TIRF lens under the control by Cell TM software (Olympus Corp., Tokyo, Japan). Images were captured by a back-illuminated Andor iXon887 EMCCD camera (512×512, 16-bit; Andor Technologies) and analyzed using MetaMorph 6.0 software.

### mGlu1α receptor-derived synthetic peptides

Peptides were synthetized to disturb the interaction between mGlu1α receptor and caveolin for Ca^2+^ imaging and co-localization study. For readily incorporation into the cells, peptides were made cell-permeable utilizing an arginine-enriched cell-membrane transduction domain of the HIV-1 Tat protein (YGRKKRRORRR) [14]. Two peptides were synthesized from AnyGen Co. Ltd. (Kwangju, Korea): Tat-blocking peptides [Tat peptide fused with caveolin binding site of mGlu1α receptor; YGRKKRRQRRR-FVTLIFVLA] and Tat-mutant peptides that do not interfere with the interaction [Tat peptide fused with caveolin binding site of mGlu1α receptor with dual point mutation; YGRKKRRQRRR-*A*VTLI*A*VLA] as a negative control. To validate the incorporation of the peptides into the plasma membrane, cells incubated with vehicle or the peptides for 45 min were stained with anti-Tat antibody (Immuno Diagnostics, MA, USA) for confocal imaging.

### Co-immunoprecipitation (Co-IP)

Co-IP was performed according to our previous procedure [16]. In brief, lysates were incubated with 2.5 μg/mL rabbit anti-caveolin or mouse anti-mGlu1α receptor (BD Bioscience) antibody for 16 h. They are then incubated with 10 μL of protein G-agarose (Santa Cruz Biotechnology, CA, USA) for 3 h at 4°C. Immunoprecipitates were extensively washed in washing buffer (25 mM Tris, pH 7.4, 10 mM NaCl, 1% Triton X-100), resuspended in 250 mM Tris, pH 6.8, 357.7 mM β-mercaptoethanol, 10% sodium dodecyl sulfate (SDS), 0.5% bromphenol blue, and 50% glycerol (5× SDS sample buffer) and then subjected to immunoblotting.

### Statistics

Data were expressed as mean ± standard error of mean (SEM). All statistical analyses were performed by Student’s t-test or one way ANOVA with post-hoc analysis where there are more than two variants using OriginPro 8 software. Statistical tests were indicated in figure legends. The differences between groups were considered to be significant when p < 0.05.

## Abbreviations

AMPA: 2-amino-3-(5-methyl-3-oxo-1,2- oxazol-4-yl) propanoic acid; DHPG: Dihydroxyphenylglycol; GM1: Monosialotetrahexosylganglioside; HEK293: Human embryonic kidney 293; IP3R: Inositol 1,4,5-triphosphate receptor; [Ca2+]c: Cytosolic Ca^2+^ level; NT solution: Normal Tyrode’s solution; MβCD: Methyl-beta-cyclodextrin; mGluR: Metabotropic glutamate receptor; MPEP: 2-methyl-6-(phenylethynyl)-pyridine; NMDA: N-Methyl-D-aspartate; PBS: Phosphate buffered saline; PFA: Paraformaldehyde; PKCα: Protein kinase Cα; PLCβ: Phospholipase C β; RFP: Red fluorescent protein; SEP: Super ecliptic pHluorin; SDS: Sodium dodecyl sulfate; TIRF: Total internal reflection fluorescence; SIM: Structured illumination microscopy.

## Competing interests

All authors declare that they have no conflict of interests.

## Authors’ contributions

SER, YHH, JK and SJK participated in the design of the study. SER and YHH carried out the molecular studies, calcium imaging, and immunoassays. DCJ participated in immunoassays. SER, YHH and SJK drafted the manuscript. All authors read and approved the final manuscript.

## Supplementary Material

Additional file 1: Figure S1 Specificity of mGlu1 receptor antibodyα and CTX-Alexa488 labeling lipid rafts in hippocampal neurons. (A) Negative control images for the specificity of mGlu1α receptor antibody. Hippocampal neurons were stained with mGlu1α receptor antibody or IgG (Red) with DAPI (Blue). Scale bar = 10 μm. The data are representative from at least 3 separate experiments. (B) Cells were labeled with CTX-Alexa 488 (green) together with antibodies recognizing transferrin receptor (negative control, red) or ganglioside GM1 (positive control, red). White boxes in the upper images are enlarged in lower images. Overlapping region (yellow) shows co-localization of green and red signals. Scale bar = 10 μm. Quantification of co-localization was presented on right. The data is shown as mean ± SEM. **Figure S2.** Tat-blocking peptides disturb mGlu1 receptor–caveolin interaction and affect mGlu1 receptor-mediated Ca^2+^ transients in HEK293 cells. (A) Co-immunoprecipitation (Co-IP) of mGlu1α receptor with caveolin in cells treated with Tat peptides (10 μM for 45 min) is shown. The Co-IP of mGlu1α receptor with caveolin was significantly reduced by Tat-blocking peptide but not by Tat-mutant peptide (n=3). (B) Effects of Tat-peptides on the intracellular Ca^2+^ transients induced by DHPG. HEK293 cells transfected with RFP-mGlu1α receptor construct were incubated with Tat-blocking/mutant peptides and loaded with Fura-2/AM. Cells were perfused with of DHPG (50 μM for 60 s). Arrows indicate the DHPG applications. n = 16 (Control), 30 (Tat-blocking peptide), 12 (Tat-mutant peptide). The data is shown as mean ± SEM from at least independent three experiments. Click here for file

## References

[B1] Nakanishi S Metabotropic glutamate receptors: synaptic transmission, modulation, and plasticity Neuron 1994 13 5 1031 1037 10.1016/0896-6273(94)90043-4 7946343

[B2] Conn PJ Pin JP Pharmacology and functions of metabotropic glutamate receptors Annu Rev Pharmacol Toxicol 1997 37 205 237 10.1146/annurev.pharmtox.37.1.205 9131252

[B3] Ferraguti F Shigemoto R Metabotropic glutamate receptors Cell Tissue Res 2006 326 2 483 504 10.1007/s00441-006-0266-5 16847639

[B4] Pin JP Duvoisin R The metabotropic glutamate receptors: structure and functions Neuropharmacology 1995 34 1 1 26 10.1016/0028-3908(94)00129-G 7623957

[B5] Hermans E Challiss RA Structural, signalling and regulatory properties of the group I metabotropic glutamate receptors: prototypic family C G-protein-coupled receptors Biochem J 2001 359 Pt 3 465 484 11672421 10.1042/0264-6021:3590465 PMC1222168

[B6] Bortolotto ZA Collingridge GL Characterisation of LTP induced by the activation of glutamate metabotropic receptors in area CA1 of the hippocampus Neuropharmacology 1993 32 1 1 9 10.1016/0028-3908(93)90123-K 8381524

[B7] Topolnik L Azzi M Morin F Kougioumoutzakis A Lacaille JC mGluR1/5 subtype-specific calcium signalling and induction of long-term potentiation in rat hippocampal oriens/alveus interneurones The Journal of physiology 2006 575 Pt 1 115 131 16740609 10.1113/jphysiol.2006.112896 PMC1819425

[B8] Allen JA Halverson-Tamboli RA Rasenick MM Lipid raft microdomains and neurotransmitter signalling Nat Rev Neurosci 2006 8 2 128 140 17195035 10.1038/nrn2059

[B9] Isshiki M Anderson RG Calcium signal transduction from caveolae Cell Calcium 1999 26 5 201 208 10.1054/ceca.1999.0073 10643558

[B10] Pani B Singh BB Lipid rafts/caveolae as microdomains of calcium signaling Cell Calcium 2009 45 6 625 633 10.1016/j.ceca.2009.02.009 19324409 PMC2695836

[B11] Besshoh S Bawa D Teves L Wallace MC Gurd JW Increased phosphorylation and redistribution of NMDA receptors between synaptic lipid rafts and post-synaptic densities following transient global ischemia in the rat brain J Neurochem 2005 93 1 186 194 10.1111/j.1471-4159.2004.03009.x 15773918

[B12] Delint-Ramirez I Salcedo-Tello P Bermudez-Rattoni F Spatial memory formation induces recruitment of NMDA receptor and PSD-95 to synaptic lipid rafts J Neurochem 2008 106 4 1658 1668 10.1111/j.1471-4159.2008.05523.x 18700282

[B13] Hou Q Huang Y Amato S Snyder SH Huganir RL Man HY Regulation of AMPA receptor localization in lipid rafts Mol Cell Neurosci 2008 38 2 213 223 10.1016/j.mcn.2008.02.010 18411055 PMC2734417

[B14] Swanwick CC Shapiro ME Yi Z Chang K Wenthold RJ NMDA receptors interact with flotillin-1 and -2, lipid raft-associated proteins FEBS Lett 2009 583 8 1226 1230 10.1016/j.febslet.2009.03.017 19298817

[B15] Hering H Lin CC Sheng M Lipid rafts in the maintenance of synapses, dendritic spines, and surface AMPA receptor stability J Neurosci 2003 23 8 3262 3271 12716933 10.1523/JNEUROSCI.23-08-03262.2003 PMC6742299

[B16] Hong YH Kim JY Lee JH Chae HG Jang SS Jeon JH Kim CH Kim J Kim SJ Agonist-induced internalization of mGluR1alpha is mediated by caveolin J Neurosci 2009 111 1 61 71 10.1111/j.1471-4159.2009.06289.x 19627451

[B17] Francesconi A Kumari R Zukin RS Regulation of group I metabotropic glutamate receptor trafficking and signaling by the caveolar/lipid raft pathway J Neurosci 2009 29 11 3590 3602 10.1523/JNEUROSCI.5824-08.2009 19295163 PMC2734995

[B18] Takayasu Y Takeuchi K Kumari R Bennett MVL Zukin RS Francesconi A Caveolin-1 knockout mice exhibit impaired induction of mGluR-dependent long-term depression at CA3-CA1 synapses Proc Natl Acad Sci 2010 107 50 21778 21783 10.1073/pnas.1015553107 21098662 PMC3003045

[B19] Shigemoto R Nakanishi S Mizuno N Distribution of the mRNA for a metabotropic glutamate receptor (mGluR1) in the central nervous system: an in situ hybridization study in adult and developing rat J Comp Neurol 1992 322 1 121 135 10.1002/cne.903220110 1430307

[B20] Shigemoto R Nomura S Ohishi H Sugihara H Nakanishi S Mizuno N Immunohistochemical localization of a metabotropic glutamate receptor, mGluR5, in the rat brain Neurosci Lett 1993 163 1 53 57 10.1016/0304-3940(93)90227-C 8295733

[B21] Kilsdonk EP Yancey PG Stoudt GW Bangerter FW Johnson WJ Phillips MC Rothblat GH Cellular cholesterol efflux mediated by cyclodextrins J Biol Chem 1995 270 29 17250 17256 10.1074/jbc.270.29.17250 7615524

[B22] Atger VM de la Llera MM Stoudt GW Rodrigueza WV Phillips MC Rothblat GH Cyclodextrins as catalysts for the removal of cholesterol from macrophage foam cells J Clin Invest 1997 99 4 773 780 10.1172/JCI119223 9045882 PMC507862

[B23] Christian AE Haynes MP Phillips MC Rothblat GH Use of cyclodextrins for manipulating cellular cholesterol content J Lipid Res 1997 38 11 2264 2272 9392424

[B24] Ablan S Rawat SS Viard M Wang JM Puri A Blumenthal R The role of cholesterol and sphingolipids in chemokine receptor function and HIV-1 envelope glycoprotein-mediated fusion Virol J 2006 3 104 10.1186/1743-422X-3-104 17187670 PMC1769366

[B25] Launikonis BS Stephenson DG Effects of membrane cholesterol manipulation on excitation-contraction coupling in skeletal muscle of the toad J Physiol 2001 534 Pt 1 71 85 11432993 10.1111/j.1469-7793.2001.00071.x PMC2278681

[B26] Harder T Scheiffele P Verkade P Simons K Lipid domain structure of the plasma membrane revealed by patching of membrane components J Cell Biol 1998 141 4 929 942 10.1083/jcb.141.4.929 9585412 PMC2132776

[B27] Malenka RC Kauer JA Perkel DJ Nicoll RA The impact of postsynaptic calcium on synaptic transmission–its role in long-term potentiation Trends Neurosci 1989 12 11 444 450 10.1016/0166-2236(89)90094-5 2479146

[B28] Marty A The physiological role of calcium-dependent channels Trends Neurosci 1989 12 11 420 424 10.1016/0166-2236(89)90090-8 2479142

[B29] Dubinsky JM Intracellular calcium levels during the period of delayed excitotoxicity J Neurosci 1993 13 2 623 631 8093901 10.1523/JNEUROSCI.13-02-00623.1993 PMC6576661

[B30] Bezprozvanny I Calcium signaling and neurodegenerative diseases Trends Mol Med 2009 15 3 89 100 10.1016/j.molmed.2009.01.001 19230774 PMC3226745

[B31] Marambaud P Dreses-Werringloer U Vingtdeux V Calcium signaling in neurodegeneration Mol Neurodegener 2009 4 20 10.1186/1750-1326-4-20 19419557 PMC2689218

[B32] Aarts MM Tymianski M TRPM7 and ischemic CNS injury Neuroscientist 2005 11 2 116 123 10.1177/1073858404272966 15746380

[B33] Weerth SH Holtzclaw LA Russell JT Signaling proteins in raft-like microdomains are essential for Ca2+ wave propagation in glial cells Cell Calcium 2007 41 2 155 167 10.1016/j.ceca.2006.06.006 16905188

[B34] Fujimoto T Nakade S Miyawaki A Mikoshiba K Ogawa K Localization of inositol 1,4,5-trisphosphate receptor-like protein in plasmalemmal caveolae J Cell Biol 1992 119 6 1507 1513 10.1083/jcb.119.6.1507 1334960 PMC2289753

[B35] Fujimoto T Miyawaki A Mikoshiba K Inositol 1,4,5-trisphosphate receptor-like protein in plasmalemmal caveolae is linked to actin filaments J Cell Sci 1995 108 Pt 1 7 15 7738118 10.1242/jcs.108.1.7

[B36] Lockwich TP Liu X Singh BB Jadlowiec J Weiland S Ambudkar IS Assembly of Trp1 in a signaling complex associated with caveolin-scaffolding lipid raft domains J Biol Chem 2000 275 16 11934 11942 10.1074/jbc.275.16.11934 10766822

[B37] Dunphy JT Greentree WK Linder ME Enrichment of G-protein palmitoyltransferase activity in low density membranes: in vitro reconstitution of Galphai to these domains requires palmitoyltransferase activity J Biol Chem 2001 276 46 43300 43304 10.1074/jbc.M104275200 11557754

[B38] Bhatnagar A Sheffler DJ Kroeze WK Compton-Toth B Roth BL Caveolin-1 interacts with 5-HT2A serotonin receptors and profoundly modulates the signaling of selected Galphaq-coupled protein receptors J Biol Chem 2004 279 33 34614 34623 10.1074/jbc.M404673200 15190056

[B39] Eroglu C Glutamate-binding affinity of Drosophila metabotropic glutamate receptor is modulated by association with lipid rafts Proc Natl Acad Sci 2003 100 18 10219 10224 10.1073/pnas.1737042100 12923296 PMC193542

[B40] Kumari R Castillo C Francesconi A Agonist-dependent signaling by group I metabotropic glutamate receptors is regulated by association with lipid domains J Biol Chem 2013 288 44 32004 32019 10.1074/jbc.M113.475863 24045944 PMC3814796

[B41] Antion MD Hou L Wong H Hoeffer CA Klann E mGluR-dependent long-term depression is associated with increased phosphorylation of S6 and synthesis of elongation factor 1A but remains expressed in S6K-deficient mice Mol Cell Biol 2008 28 9 2996 3007 10.1128/MCB.00201-08 18316404 PMC2293080

[B42] Okamoto T Schlegel A Scherer PE Lisanti MP Caveolins, a family of scaffolding proteins for organizing “preassembled signaling complexes” at the plasma membrane J Biol Chem 1998 273 10 5419 5422 10.1074/jbc.273.10.5419 9488658

[B43] Song KS Li S Okamoto T Quilliam LA Sargiacomo M Lisanti MP Co-purification and direct interaction of Ras with caveolin, an integral membrane protein of caveolae microdomains. Detergent-free purification of caveolae microdomains J Biol Chem 1996 271 16 9690 9697 10.1074/jbc.271.16.9690 8621645

[B44] Isshiki M Ando J Korenaga R Kogo H Fujimoto T Fujita T Kamiya A Endothelial Ca2+ waves preferentially originate at specific loci in caveolin-rich cell edges Proc Natl Acad Sci U S A 1998 95 9 5009 5014 10.1073/pnas.95.9.5009 9560219 PMC20204

[B45] Bading H Ginty DD Greenberg ME Regulation of gene expression in hippocampal neurons by distinct calcium signaling pathways Science 1993 260 5105 181 186 10.1126/science.8097060 8097060

[B46] Lue LF Kuo YM Roher AE Brachova L Shen Y Sue L Beach T Kurth JH Rydel RE Rogers J Soluble amyloid beta peptide concentration as a predictor of synaptic change in Alzheimer’s disease Am J Pathol 1999 155 3 853 862 10.1016/S0002-9440(10)65184-X 10487842 PMC1866907

[B47] Li S Hong S Shepardson NE Walsh DM Shankar GM Selkoe D Soluble oligomers of amyloid β protein facilitate hippocampal long-term depression by disrupting neuronal glutamate uptake Neuron 2009 62 6 788 801 10.1016/j.neuron.2009.05.012 19555648 PMC2702854

[B48] McBride SM Choi CH Schoenfeld BP Bell AJ Liebelt DA Ferreiro D Choi RJ Hinchey P Kollaros M Terlizzi AM Pharmacological and genetic reversal of age-dependent cognitive deficits attributable to decreased presenilin function The Journal of neuroscience: the official journal of the Society for Neuroscience 2010 30 28 9510 9522 10.1523/JNEUROSCI.1017-10.2010 20631179 PMC2917645

[B49] Fundytus ME Yashpal K Chabot JG Osborne MG Lefebvre CD Dray A Henry JL Coderre TJ Knockdown of spinal metabotropic glutamate receptor 1 (mGluR(1)) alleviates pain and restores opioid efficacy after nerve injury in rats Br J Pharmacol 2001 132 1 354 367 10.1038/sj.bjp.0703810 11156596 PMC1572554

[B50] Schkeryantz JM Kingston AE Johnson MP Prospects for metabotropic glutamate 1 receptor antagonists in the treatment of neuropathic pain J Med Chem 2007 50 11 2563 2568 10.1021/jm060950g 17489573

[B51] Aiba A Kano M Chen C Stanton ME Fox GD Herrup K Zwingman TA Tonegawa S Deficient cerebellar long-term depression and impaired motor learning in mGluR1 mutant mice Cell 1994 79 2 377 388 10.1016/0092-8674(94)90205-4 7954803

[B52] Guergueltcheva V Azmanov DN Angelicheva D Smith KR Chamova T Florez L Bynevelt M Nguyen T Cherninkova S Bojinova V Autosomal-recessive congenital cerebellar ataxia is caused by mutations in metabotropic glutamate receptor 1 Am J Hum Genet 2012 91 3 553 564 10.1016/j.ajhg.2012.07.019 22901947 PMC3511982

[B53] Zhang F Liu B Lei Z Wang JH mGluR(1),5 activation improves network asynchrony and GABAergic synapse attenuation in the amygdala: implication for anxiety-like behavior in DBA/2 mice Mol Brain 2012 5 20 10.1186/1756-6606-5-20 22681774 PMC3475049

[B54] Mikulecka A Mares P Effects of mGluR5 and mGluR1 antagonists on anxiety-like behavior and learning in developing rats Behav Brain Res 2009 204 1 133 139 10.1016/j.bbr.2009.05.032 19505510

