# Supplementary material for: Lipid rafts serve as signaling platforms for mGlu1 receptor-mediated calcium signaling in association with caveolin
Source: Mol Brain. 2014 Feb 10;7:9. doi: 10.1186/1756-6606-7-9 (PMC3937055; doi:10.1186/1756-6606-7-9)
Supplement: Additional file 1: Figure S1 — Specificity of mGlu1 receptor antibodyα and CTX-Alexa488 labeling lipid rafts in hippocampal neurons. (A) Negative control images for the specificity of mGlu1α receptor antibody. Hippocampal neurons were stained with mGlu1α receptor antibody or IgG (Red) with DAPI (Blue). Scale bar = 10 μm. The data are representative from at least 3 separate experiments. (B) Cells were labeled with CTX-Alexa 488 (green) together with antibodies recognizing transferrin receptor (negative control, red) or ganglioside GM1 (positive control, red). White boxes in the upper images are enlarged in lower images. Overlapping region (yellow) shows co-localization of green and red signals. Scale bar = 10 μm. Quantification of co-localization was presented on right. The data is shown as mean ± SEM. Figure S2. Tat-blocking peptides disturb mGlu1 receptor–caveolin interaction and affect mGlu1 receptor-mediated Ca2+ transients in HEK293 cells. (A) Co-immunoprecipitation (Co-IP) of mGlu1α receptor with caveolin in cells treated with Tat peptides (10 μM for 45 min) is shown. The Co-IP of mGlu1α receptor with caveolin was significantly reduced by Tat-blocking peptide but not by Tat-mutant peptide (n=3). (B) Effects of Tat-peptides on the intracellular Ca2+ transients induced by DHPG. HEK293 cells transfected with RFP-mGlu1α receptor construct were incubated with Tat-blocking/mutant peptides and loaded with Fura-2/AM. Cells were perfused with of DHPG (50 μM for 60 s). Arrows indicate the DHPG applications. n = 16 (Control), 30 (Tat-blocking peptide), 12 (Tat-mutant peptide). The data is shown as mean ± SEM from at least independent three experiments. [file 1756-6606-7-9-S1.pptx]

## Slide 1
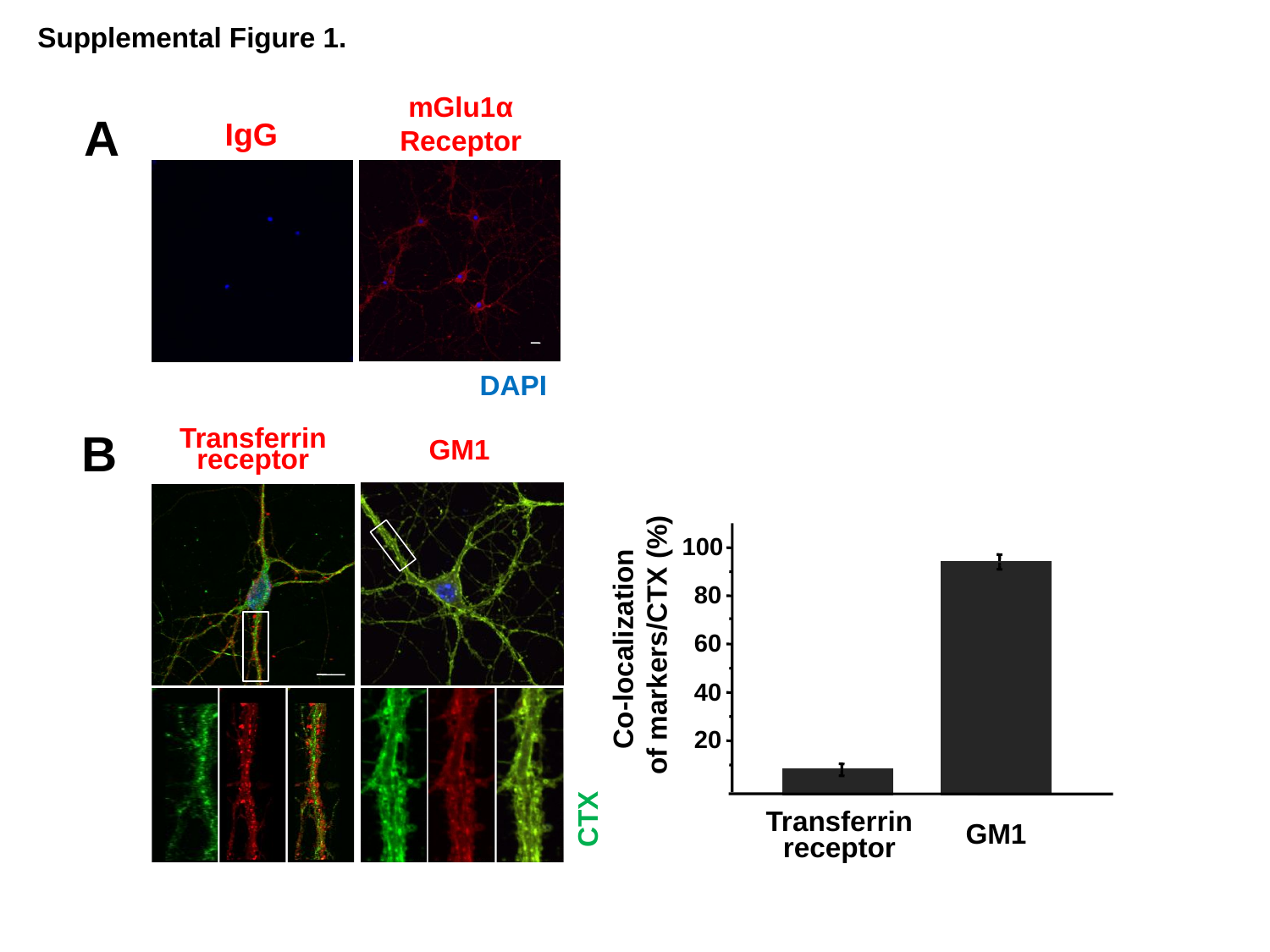

# Supplemental Figure 1.
mGlu1α
Receptor
A
IgG
DAPI
B
GM1
Transferrin
receptor
100
80
Co-localization
 of markers/CTX (%)
60
40
20
Transferrin
receptor
GM1
CTX

## Slide 2
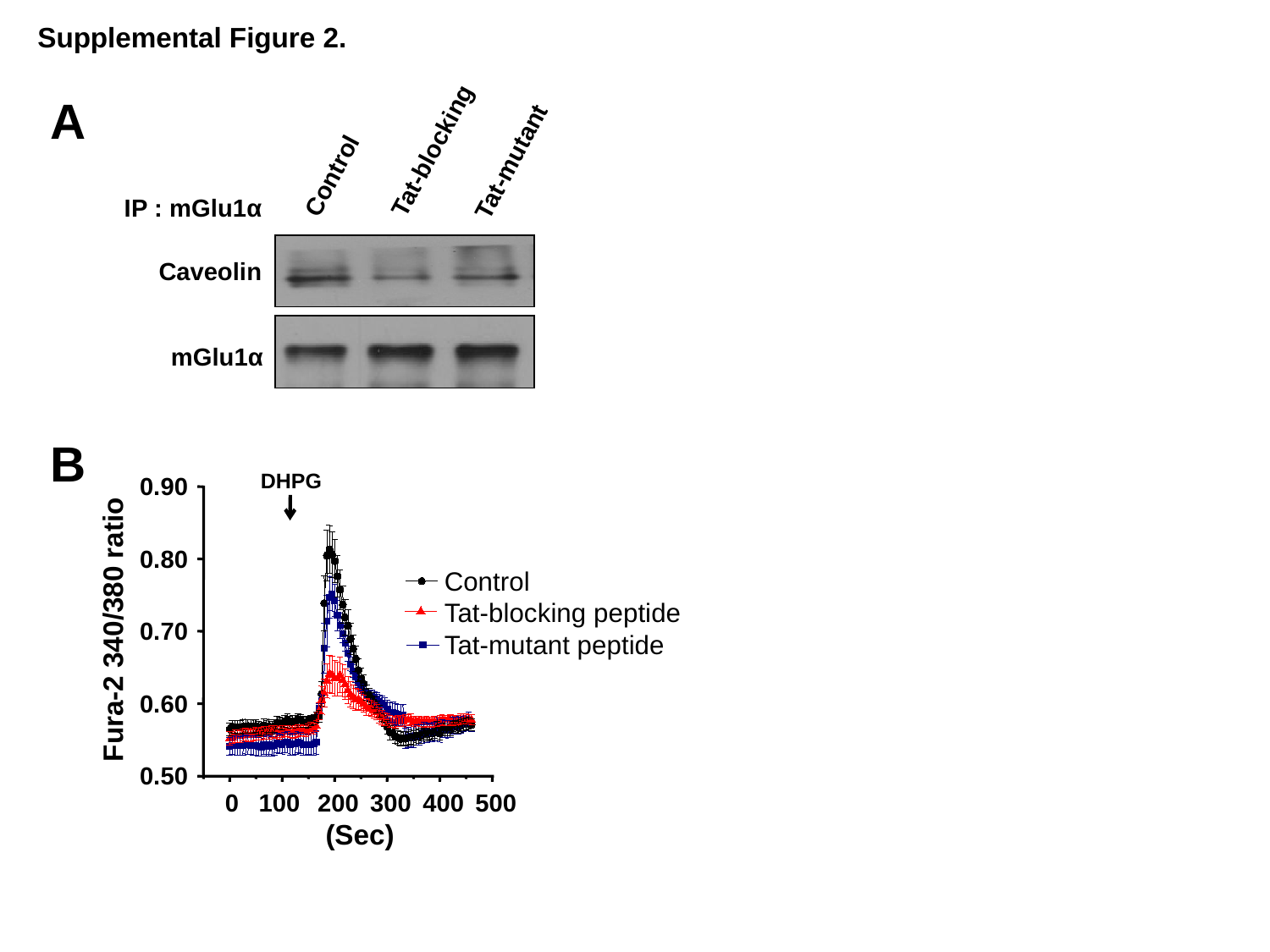

# Supplemental Figure 2.
A
Tat-blocking
Tat-mutant
Control
IP : mGlu1α
Caveolin
mGlu1α
B
DHPG
0.90
0.80
Control
Tat-blocking peptide
Tat-mutant peptide
Fura-2 340/380 ratio
0.70
0.60
0.50
0
100
200
300
400
500
(Sec)
